# The impact of early-life sub-therapeutic antibiotic treatment (STAT) on excessive weight is robust despite transfer of intestinal microbes

**DOI:** 10.1038/s41396-019-0349-4

**Published:** 2019-01-16

**Authors:** Anjelique F. Schulfer, Jonas Schluter, Yilong Zhang, Quincy Brown, Wimal Pathmasiri, Susan McRitchie, Susan Sumner, Huilin Li, Joao B. Xavier, Martin J. Blaser

**Affiliations:** 1 Department of Medicine, New York University Langone Medical Center, New York, NY 10016 USA; 2 Computational Biology Program, Memorial Sloan-Kettering Cancer Center, New York, NY 10065 USA; 3 Department of Population Health, New York University Langone Medical Center, New York, NY 10016 USA; 4 Eastern Regional Comprehensive Metabolomics Resource Core, University of North Carolina at Chapel Hill, Chapel Hill, NC USA; 5 New York Harbor Veterans Affairs Medical Center, New York, NY 10010 USA

**Keywords:** Microbiology, Health care

## Abstract

The high-fat, high-calorie diets of westernized cultures contribute to the global obesity epidemic, and early life exposure to antibiotics may potentiate those dietary effects. Previous experiments with mice had shown that sub-therapeutic antibiotic treatment (STAT)—even restricted to early life—affected the gut microbiota, altered host metabolism, and increased adiposity throughout the lifetime of the animals. Here we carried out a large-scale cohousing experiment to investigate whether cohousing STAT and untreated (Control) mice would transfer the STAT-perturbed microbiota and transmit its impact on weight. We exposed pregnant dams and their young offspring to either low-dose penicillin (STAT) or water (Control) until weaning, and then followed the offspring as they grew and endured a switch from normal to high-fat diet at week 17 of life. Cohousing, which started at week 4, rapidly approximated the microbiota within cages, lowering the weight of STAT mice relative to non-cohoused mice. The effect, however, varied between cages, and was restricted to the first 16 weeks when diet consisted of normal chow. Once mice switched to high-fat diet, the microbiota α- and β-diversity expanded and the effect of cohousing faded: STAT mice, again, were heavier than control mice independently of cohousing. Metabolomics revealed serum metabolites associated with STAT exposure, but no significant differences were detected in glucose or insulin tolerance. Our results show that cohousing can partly ameliorate the impact of STAT on the gut microbiota but not prevent increased weight with high-fat diet. These observations have implications for microbiota therapies aimed to resolve the collateral damage of antibiotics and their load on human obesity.

## Introduction

Diet, lifestyle, and human genetics do not sufficiently explain the rapid global obesity increase [1–3]. Further studies are necessary to understand the causes of this pandemic and to curtail its trajectory. The composition of the intestinal microbiota influences how its host gains weight. Experiments with mice showed how the microbiota influences energy storage and metabolism [4–9]. As learned decades ago, germ-free mice, which lack a gut microbiota, remain leaner than mice with a conventional microbiota even when fed a high-fat diet; this indicates the essential role of the microbiota in efficient energy utilization [6, 10]. The composition of the gut microbiota partially determines how the host harvests energy from food and absorbs nutrients in the intestine, among other systemic processes [4, 5, 7, 8]. Therefore, changing the composition of the gut microbiota could influence the propensity for obesity.

Antibiotics are essential therapeutic agents to fight pathogenic bacteria but they also impact the commensal bacteria that comprise the gut microbiota [11–14]. The damage caused by antibiotics depends on their mechanism of action, dosage, treatment duration, and administration route. Antibiotics given at low doses to animals have the notable effect of increasing weight—a practice termed sub-therapeutic antibiotic treatment (STAT) and used since 1946 in livestock [15]. Experiments showing that the effect of STAT is lost in germ-free chicks indicated, more than 50 years ago, that the way antibiotics increase growth depends on animals having a gut microbiota [16]. STAT using essentially any anti-bacterial agent causes weight gain, and has been routinely practiced in livestock husbandry because it increases meat production yields [17]. Whether STAT also causes weight gain in humans is unclear. Trials conducted between the 1950s and 1970s sought to determine whether antibiotic supplementation could be used to treat human malnutrition; however, the trials were halted because of concerns about selecting for antibiotic-resistant bacteria [18]. By 2005, the alarming global rise in obesity brought attention back to STAT, now as a possible cause of excessive weight gain due to unintended exposures [19].

More recently, experiments in mice, in conjunction with high-throughput DNA sequencing, provided definite evidence that STAT can alter the gut microbiota and increase adiposity [20]. Strikingly, STAT effects were most profound and durable when the antibiotic exposure started early in life [21], as done on farms. The microbiota composition recovered over time after ending the antibiotic exposure, but the mice continued to be more adipose. Early life is a critical developmental window in both mice and humans [22, 23] and perturbing the microbiota during that time may have long-term consequences for obesity in humans as well. It remains to be tested whether recovering the microbiota prevents those long-term consequences or could ameliorate obesity.

The many links between the gut microbiota, health, and a wide range of diseases beyond obesity—including autism, inflammatory bowel disease (IBD), and cancers—make engineering the microbiota an appealing goal [24]. Prebiotics, probiotics, antibiotics, and genetically modified bacteria provide possible ways to tailor the microbiota. Since mice are coprophagic, cohousing can be conveniently used as a means to influence gut microbiota composition and study its effect on host phenotypes [25, 26].

Here, we conducted a large-scale cohousing experiment to determine how microbes transmitted between STAT and untreated mice might alter the impact of early-life STAT on weight gained throughout life. Our results illuminate a complex interplay between early-life STAT, the gut microbiota and its host’s response to lifetime dietary changes and constitute a sizeable dataset that can be further mined to investigate the role of microbiota transmission on host weight—one of the most well-characterized and reproducible phenotypes linked to microbiota composition.

## Results

### Cohousing ameliorates the impact of STAT on weight in chow but not high-fat diet

**Fig. 1:**
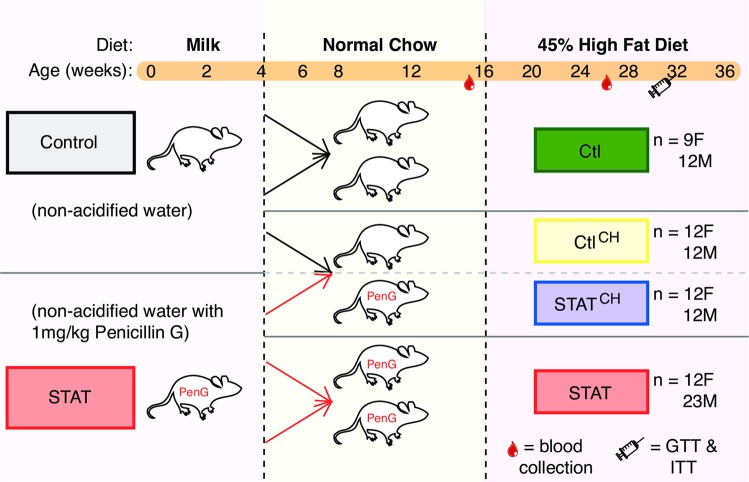
Summary scheme for cohousing experiment (see Fig. S1 for further details). Dams (*n* = 20) were bred and randomly assigned to become Control (water) or STAT (1 mg/kg body weight penicillin G) when pregnancy was detected. The antibiotic treatment continued until their 105 pups were weaned at week 4. Of these pups, 57 were not cohoused and 48 were used for cohousing. After a 3-day wash-out period, mice were either litter-mixed within treatment (non-cohoused) or mixed at a 1:1 ratio of STAT:Control within one cage (cohoused). The mice were changed to 45% high-fat diet at week 16. Fecal pellets and scale weight were collected 1–5 times/week starting at week 3. We also collected blood at ~16 and 26 weeks and performed two glucose tolerance tests, with the final test having a paired insulin tolerance test

We had previously shown that early-life STAT, i.e. treatment limited to the first 4 weeks of life, was sufficient to increase weight gain throughout life [21]. Here we carried out a large-scale experiment with 105 mice to determine the impact of cohousing. All mice started with regular chow and switched to a high-fat diet later in life (Fig. 1).

More specifically, we bred 20 C57BL/6 mice and, when pregnancy was detected, we randomly assigned those 20 females to Control (normal water) or STAT (water with penicillin G at 1 mg/kg body weight). The STAT (or not) continued until the 105 pups were 4 weeks old. Pups had access to both the dams’ water (with penicillin G in the case of STAT) and diet (normal chow) until week 4; this was the only time STAT mice were exposed to any antibiotic. Then, the pups switched to different cages where they continued to receive normal chow until week 16. The cages were set up such that some mice resided with mice that received the same treatment and these were termed non-cohoused mice; other mice were placed in cages with four mice each where two were Control mice and two were STAT mice but always of the same sex and different litters (see Fig S1 for the full arrangement of the 105 mice according to their litters and cages). After week 16, the mice were switched to a 45% high-fat diet and were followed until the experiment ended on week 36.

**Fig. 2:**
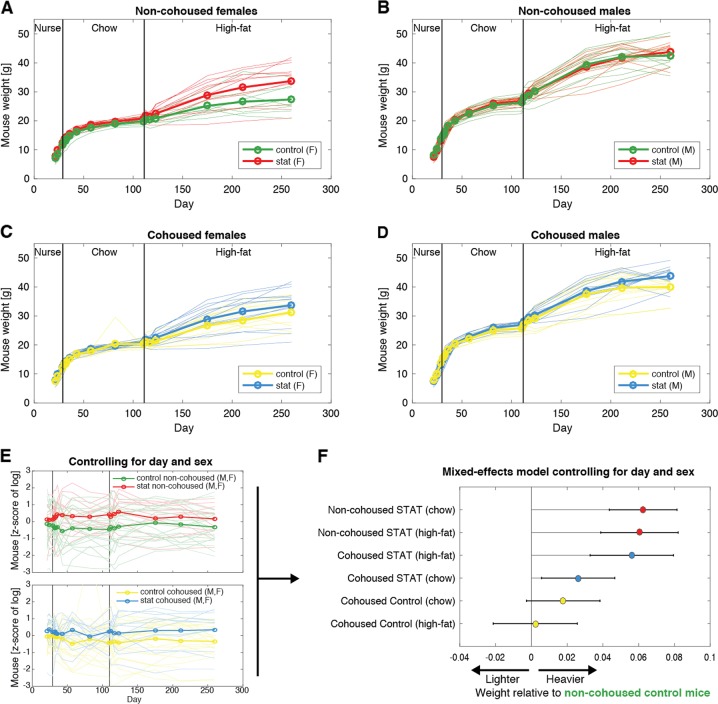
STAT increases weight; cohousing transmits that effect but only in chow diet. **a**–**d** Trajectories of mouse weight through life, stratified by sex and the experimental variable (STAT vs Control, cohoused vs non-cohoused). **e** Weights were log-transformed and then considered in a mixed effects model that controlled for day and sex. This plot illustrates the compensation for sex and day, but the model incorporated that compensation directly as random effects, obviating the need for a *z*-score transformation in the response variable (log-transformed weight). **f** The effects determined for the impact of the STAT and cohousing on the log-transformed weight show that STAT increases weight, and cohousing transmits that effect but only in chow diet. The scale of the effects is in multiples of the average weight (e.g. 0.1 represents an average weight that is 0.1×, or 10%, heavier)

Mice gained weight to ~15–18 g in the 4 weeks of nursing. Growth slowed during the 12 weeks of chow diet during which mice grew to ~20–30 g. Then, they gained weight faster again when they were switched to high-fat diet for the last 20 weeks of the experiment; they reached a final average weight of 37 g, but with substantial variability between mice (Fig. 2a–d).

We analyzed how our experimental variables, STAT and cohousing, impacted the weight of mice compared to the Control non-cohoused mice. We employed a linear mixed effects model that accounted for the increasing average weights over time and, importantly, accounted for sex as a biological variable (Fig. 2e). This was necessary because males were on average ~30% heavier than females (*P* « 0.01). According to this model and consistent with our prior studies [20, 21, 27], STAT non-cohoused mice were on average ~6% heavier than the reference group of Control non-cohoused mice of the same sex (*P* « 0.01), and we now show that this effect was the same during the chow and high-fat diet periods (Fig. 2f). Cohousing partly ameliorated the impact of STAT but only during the chow diet period: during chow, STAT mice cohoused with Control mice were heavier than the reference group by 2.6% (*P* = 0.012), but during the high-fat diet, STAT cohoused mice were even heavier, 5.6% compared to the reference Control non-cohoused mice (*P* « 0.01), which was indistinguishable from the ~6% heavier weight of STAT non-cohoused mice. Control mice cohoused with STAT were marginally heavier than Control non-cohoused mice during chow (1.8%, *P* = 0.09). Their weight was not distinguishable from that of Control non-cohoused mice during the high-fat diet (0.2%, *P* = 0.86).

Those results suggested that cohousing partially ameliorated the effect of STAT but only during chow. To confirm this, we repeated the analysis switching the reference group so that the reference was now STAT non-cohoused mice. This model showed that during the chow diet, cohoused STAT mice were 3.8% lighter than that reference (*P* = 3 × 10^−5^), but there was no significant difference during high-fat diet (*P* = 0.6). We also asked directly whether the interaction between cohousing and diet was significant in STAT mice by building a mixed effects model with an interaction term. This model determined that the interaction was indeed significant (*P* = 0.036), again confirming that cohousing ameliorated the effect of STAT but only during the chow period.

In summary, our data confirmed that early life STAT increases weight throughout life [21] and showed that cohousing could partially ameliorate that effect, at least transiently, highlighting the role of phenotype-changing gut microbes that may have been exchanged through coprophagy. The impact of cohousing, however, was observed only during the chow diet, because the weight-enhancing effect of early life STAT returned once mice switched to high-fat diet. Control cohoused mice were indistinguishable from their non-cohoused counterparts (Fig. 2f).

**Fig. 3:**
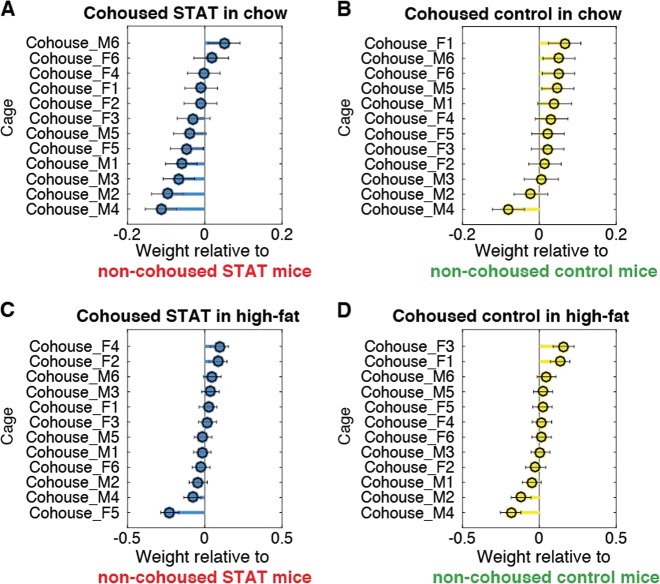
The impact of cohousing on the weight of STAT and Control mice shows a clearer trend in chow than in high-fat diet, but differs significantly between cages. Panels **a–d** show effects determined for the indicated experimental variables using a linear mixed effects model. The asymmetric distribution of predominantly negative effects in panel **a** shows that STAT mice cohoused with Control mice more often weighed less than their comparison group (i.e. STAT mice living with other STAT mice); similarly, the asymmetric distribution of effects in panel **b** shows that Control mice in cages cohoused with STAT were predominantly heavier than their comparison group (Control mice living with other Control mice, i.e. non-cohoused). The asymmetry, and thus the effect of cohousing on the average phenotype, was mostly lost when mice received the high-fat diet (**c**, **d**). The scale of the effects is in multiples of the average weight (e.g. 0.1 represents an average weight that is 0.1×, or 10%, heavier)

When we investigated each cohousing cage separately (Fig. 3), we saw that the weight-reducing effect of cohousing on STAT mice was significant (*P* < 0.05) in 5 of 12 cages (Fig. 3a). Similarly, the weight-increasing effect of cohousing in Control mice was significant in only 4 of 14 cages (Fig. 3b). Therefore, the weight effects varied among cages. Nonetheless, this analysis confirmed that the impact of cohousing in ameliorating the effect of STAT was more pronounced during the chow diet phase (Fig. 3a) and seemed to vanish during high-fat diet (Fig. 3d).

### The effect of STAT and cohousing on blood metabolites in female mice

Previous results had shown that STAT impacts females more than males; females gained more weight and accumulated more adipose tissue due to STAT [21, 27]. In this study non-cohoused mice can be used to verify such prior findings: inspection of the trends (compare Figs. 2a, b) immediately suggests that females gained more weight during high-fat diet due to STAT than did males. To more formally investigate the interactions between STAT, cohousing, diet, and sex, we used a mixed effects model. Application of this model supported that the impact of STAT on females was stronger (Fig S2).

Therefore, we chose to focus on female mice for metabolomics analyses. We measured serum metabolites in female mice at week 15, just before the switch to high-fat diet, to search for compounds that might contribute to each group’s differential weight gain. We used gas chromatography/time-of-flight mass spectrometry (GC/TOF MS) to measure a panel of metabolites (Fig S3) and applied a supervised statistical analysis—orthogonal partial least-squares discriminant analysis (OPLS-DA [28])—to determine the set of metabolites that differed consistently among all four groups (Fig S3A–E). The analysis revealed small changes in many metabolites, many unidentified but some known (Fig S3F). Among those identified, in STAT and in Control cohoused females, there were higher levels of cellobiose and vitamin E (alpha-tocopherol) compared to Control non-cohoused females; these differences were consistent with the roles of these metabolites in the more rapid weight gain during high-fat diet. Levels of citric acid, 2-hydroxybutanoic acid, and phenylalanine were higher in each cohousing group compared to their non-cohoused counterpart, and stearic acid was lower, suggesting roles in the synergistic growth effects of cohousing for both Control and STAT mice (Fig S3F).

In previous studies, lifelong STAT had increased the incidence and severity of metabolic abnormalities, including glucose intolerance, increased hepatic lipids, and histologic steatosis [27]. However, here these effects were not observed, possibly due to the limited (4-week) STAT exposure: 31-week-old mice showed no significant differences in glucose or insulin tolerance due to STAT in either female or male mice, nor obvious steatosis (Fig S4).

### Cohousing mixes microbiota and transfers impact of STAT during normal chow diet

**Fig. 4:**
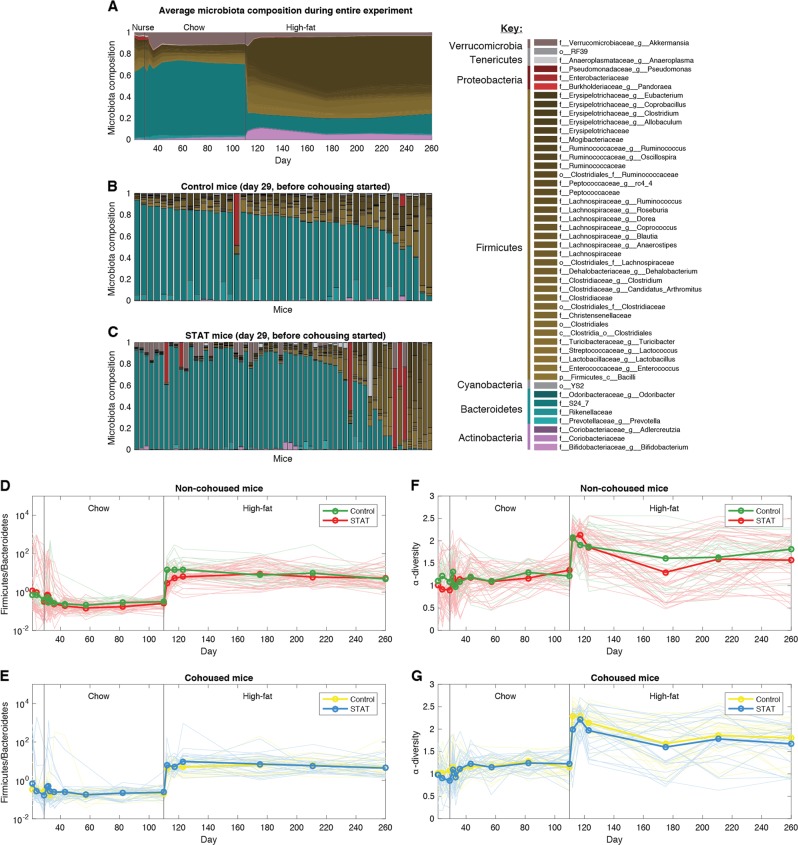
The shift to high-fat diet increased Firmicutes and α-diversity, and had the strongest impact on microbiota composition. **a** The average composition across all mice changed from a microbiota dominated by Bacteroidetes (in blue) to one dominated by Firmicutes (in brown). **b**, **c** The compositions in each mice at the last day of nursing, before the cohousing started. The Control and STAT mice are ranked, from left to right, by their ratio of Firmicutes to Bacteroidetes. **d**, **e** Ratio of Firmicutes to Bacteroidetes, a quantity previously linked to high-fat diet and obesity, increased in high-fat diet, but STAT had no significant impact. **f**, **g** The α-diversity (calculated using the Shannon index) also increased when high-fat diet was begun but STAT had no significant impact

To further dissect the complex interplay of STAT and cohousing, we investigated the composition of the gut microbiota and how it changed in each of the 105 studied mice. We determined compositions of 1741 fecal samples collected over time for the 105 mice using 16S rRNA amplicon sequencing. The average composition fluctuated significantly during the nursing period, but then stabilized once mice were weaned and begun on chow diet for the cohousing period (Fig. 4a). The most abundant bacteria during those 12 weeks of chow belonged to S24-7, a family of the Bacteroidetes phylum which represented >60% of the average microbiota. *Akkermansia muciniphila*, the second most abundant taxon, represented ~10%.

Before cohousing started there was substantial variability in the microbiota composition of Control mice (Fig. 4b); STAT mice had also large variability (Fig. 4c), but compared to Control, more mice had blooms in Proteobacteria (in red) and *Akkermansia* (gray).

The strongest factor shaping the microbiota was the shift to high-fat diet, which resulted in an abrupt compositional change at day 110 (Fig. 4a). The microbiota then stabilized rapidly in the next 10 days to a composition where microbes in genus *Allobaculum* of the phylum Firmicutes rose in abundance; S24-7 decreased to ~14%. The ratio of Firmicutes to Bacteroidetes, a compositional metric associated with high-fat diet and obesity, and linked to high-fat diet [7], increased, consistent with those prior studies (Fig. 4d, e). Neither STAT nor cohousing had any appreciable impact on the ratio of Firmicutes to Bacteroidetes beyond nursing.

### Microbiota ecology revealed profound changes caused by high-fat diet, and the influences of STAT and cohousing

The large variability in microbiota composition between individual mice (Fig. 4a, b) led us to mathematical ecology to assess the impact of diet, STAT, and cohousing on the microbiota. We first quantified the α-diversity using the Shannon index. The α-diversity of each sample fluctuated substantially during nursing, then stabilizing to an average of ~1.1 during chow, with values varying widely between ~0.5 and 1.7 across all mice. High-fat diet increased the average α-diversity to ~1.8, with values varying between ~0.7 and 2.7 in mice. STAT and cohousing had no visible impact on the average α-diversity during chow or high-fat diet (Fig. 4f, g).

**Fig. 5:**
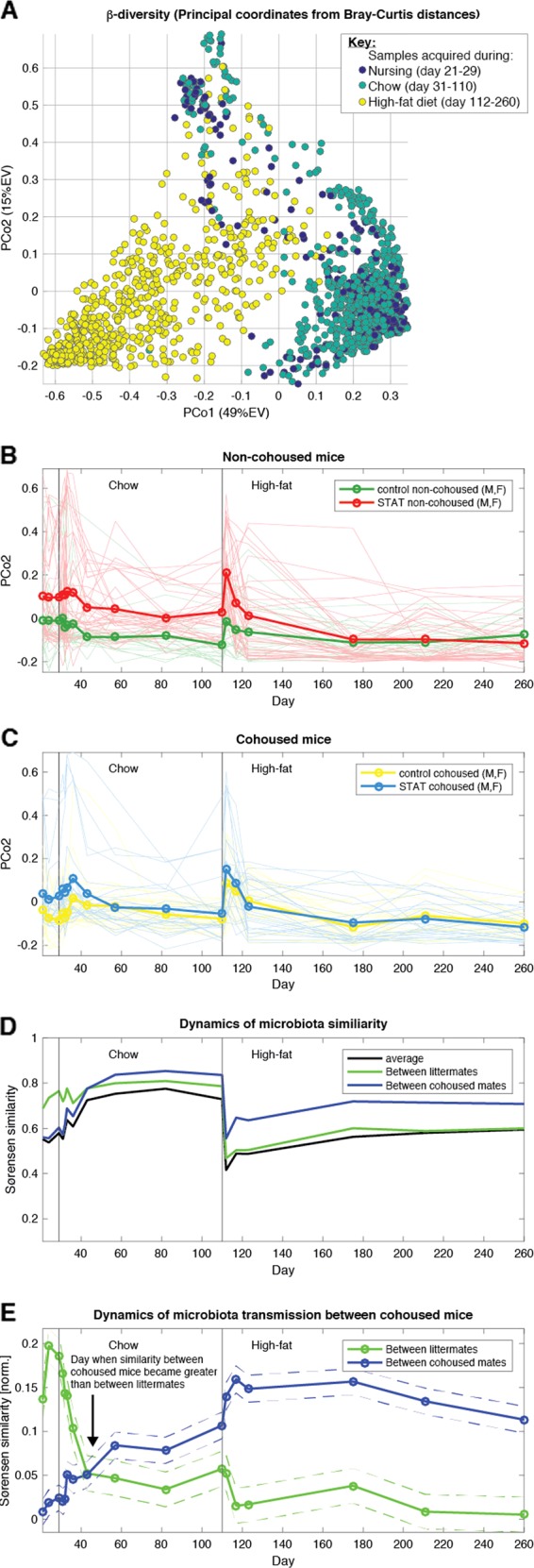
Cohousing mixed the microbiota and ameliorated the impact of STAT on composition, but high-fat diet quickly diversified the microbiota. **a** β-Diversity investigated using principal coordinate analysis of Bray–Curtis distances between samples captured the effect of diet in the first coordinate (PCo1, 49% explained variance (EV)). **b** Time series of PCo2 (15% EV) reveals that it captures—at least partially—the effect of STAT on the microbiota. **c** The effect of STAT is ameliorated in cohoused mice. **d** Analysis of between-mouse similarity (Sørensen similarity index) shows that the microbiota, which start the cohousing period dissimilar, become more similar during the chow period. The similarity temporarily plummets during the rapid microbiota shifts caused by the switch to high-fat diet. **e** A detailed analysis of the Sørensen similarity pinpoints the exact time when mice became more similar to their cohousing mates compared to their littermates: after 2 weeks of cohousing (cohousing started with chow, the first vertical line). The microbiota similarity-increasing effect of cohousing became more pronounced in high-fat diet, while the average similarity between all mice plummeted simultaneously. For each time point, we modeled *similarity ~ Cage* *+* *Litter* (see Methods). Dashed lines show the intervals determined by the standard error of the parameter estimates

The β-diversity computed from the Bray–Curtis distances and their principal coordinate analysis revealed that the first two principal coordinates captured most of the variance among the microbiota samples. PCo1 (49% EV) captured the drastic change from chow to high-fat diet (Fig. 5a). PCo2, less pronounced (15% EV), was higher in STAT non-cohoused mice compared to Control non-cohoused mice, especially during chow diet (Fig. 5b). The link between STAT and PCo2 was dampened in cohoused mice, and was higher in Control cohoused mice compared to their non-cohoused counterparts (Fig. 5c, see also Fig. S5). Therefore, PCo2 captured parts of the impact of STAT on the microbiota composition and its transmissibility between cohoused mice.

To quantify how the similarity between mice changed over time, we calculated the Sørensen similarity index (SSi), obtained directly from the Bray–Curtis distance (BC) using the equation: SSi = 1–BC. The analysis revealed that the average microbiota similarity—calculated across all pairs of mice—increased as the mice passed through the chow period (Fig. 5d, black line). Cohoused mice were more similar (Fig. 5d, blue line), as expected, since mice may transmit microbes by coprophagy.

The similarity plummeted when the mice switched to high-fat diet, indicating that the sudden shift to a Firmicutes-dominated microbiota with greater α-diversity (Fig. 4) led to divergences of the microbiota compositions of different mice. Compositional divergence was less among cohoused mice, but remained significant compared to pre-high-fat (Fig. 5d, blue line). By that point, the similarity between original littermates was as low as the average similarity between mice (Fig. 5d, green line), confirming that cohousing has a strong effect homogenizing the microbiota composition. Nonetheless, high-fat diet switch had strong effects; the divergences indicated that the microbiota became more individualized, with potential implications for the host’s weight change.

### Cohousing takes approximately 2 weeks to transmit the intestinal microbiota

We next asked whether we could determine the rapidity of the microbiota transmission between cohoused mice. We used a linear regression to compare, across time, the similarity between cohoused mice with the similarity of original littermates. As expected, before cohousing the microbiota was most similar between littermates (Fig. 5e, green line). The similarity between littermates plunged as soon as cohousing started, and after 2 weeks of cohousing, the compositions already were more similar between cohoused mates (Fig. 5e, blue line). The similarities between cohoused mice had decreased when the mice were shifted to the high-fat diet, but the similarities between mice housed in different cages fell more as the total diversity in the experiment increased. The relative importance of cohousing on within-cage similarity relative to all other mice appeared to be even stronger during high-fat diet (Fig. 5e, blue line compared to green line).

### Microbial taxa linked to weight identified in chow and high-fat diet

Thus far, the ecological analyses provided evidence that cohousing led to microbiota composition convergence among mice housed within the same cage, and increased the differences relative to littermates that had received the same treatment. That effect of cohousing on the microbiota was detectable during the chow and high-fat diet periods (Fig. 2d). However, the effect in high-fat diet contrasted with our earlier finding that cohousing impacted weight during the chow period but not during the high-fat diet period (Fig. 2e). The higher diversity that came with the switch to high-fat diet could underlie this: high-fat diet increased both the α-diversity (diversity within a mouse, Fig. 4f, g) as well as the β-diversity (among pairs of mice, Fig. 5d). The compositions of different mice diverged, even though they diverged slightly less for those within the same cage (Fig. 5e); this process of microbiota individualization could be obscuring the link between STAT, microbiota composition, and weight.

We turned to a supervised machine learning approach to identify the taxa most strongly associated with weight variation during the different diets. We employed an elastic net regression with five-fold cross-validation and a stratification scheme that considered both diet and sex. The regression was run separately twice, to identify the microbes associated with weight during chow (first run) and during high-fat diet (second). We ran two separate regressions since we expected distinct associations between microbes and weight depending on the diet [29].

**Fig. 6:**
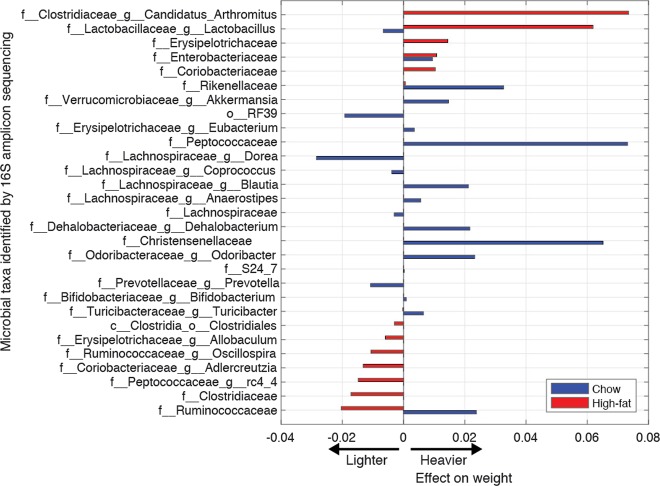
Supervised learning reveals links between microbes and weight in chow and high-fat diet. Two elastic net regressions run separately for chow and high-fat diet identified microbes that correlated with *z*-score (per sex, each day) of the log-transformed weight. Microbes that passed the stringent cross-validation are shown ranked by their association with weight in high-fat diet

The analysis revealed 20 microbes with a strong link to weight during the chow diet (Fig. 6, blue). Among those most associated with higher weight was the family Christensenellaceae, predominant in STAT non-cohoused mice but whose levels were relatively lower in Control mice and in STAT cohoused mice, frequencies consistent with a transmissible phenotype.

The separate analysis conducted for the high-fat diet period identified 14 microbes with a strong link to weight (Fig. 6, red). As expected, these 14 microbes were most often distinct from the 20 microbes found for chow—with the exception of the family Enterobacteriaceae, linked to heavier weight in both diets. Some taxa showed significant opposing effects in the two diets: The genus *Lactobacillus* was associated with lighter weight in chow but heavier weight in high-fat diet; this inversion was notable because *Lactobacillus* was decreased in STAT mice until the beginning of the chow diet, but then had increased in STAT mice at the beginning of the high-fat diet—a period when the mice gained weight rapidly. The family Ruminococcaceae was unique in being linked to heavier weight in chow but lighter weight during the high-fat diet period. Notably, these bacteria were less abundant in STAT mice during the high-fat diet, even for those mice cohoused with Controls. The Ruminococcaceae were consistent for microbes that contributed to lower weight in high-fat; its lower abundance in STAT mice—regardless of cohousing—could explain the heavier weight of those animals.

## Discussion

The results we present here show that the impact of early-life STAT on weight during high-fat diet is robust even in the face of transfer of gut microbiota by cohousing. STAT exposure increases weight of mice and their adiposity later in life [20], similar to the effect of antibiotics used in livestock [15, 17]. The effect occurs even when the antibiotic is limited to early life [21] and as shown here, can withstand the mixing of microbiota occurring through coprophagy by cohousing STAT-exposed and non-exposed mice.

The effect of cohousing on approximating the microbiota composition of mice residing in the same cage occurred within 2 weeks. The switch to high-fat diet, however, caused an even more rapid change in microbiota composition from a Bacteroidetes-dominated community to one that was Firmicutes-dominated. The average α-diversity practically doubled during this transition. This change in community composition and the rapid diversification dominated the effect of cohousing, with STAT mice becoming on average heavier than Control mice regardless of cohousing.

Previous studies have characterized links between microbiota diversity and high-fat diet, but the direction of the association has not always been consistent: high-fat diet has decreased [30] or increased α-diversity [31, 32]. Here, the switch to high-fat diet clearly increased both α-diversity and β-diversity. This result suggests that the diversity was limited by conditions provided by the chow diet, such as a high-fiber environment favoring Bacteroidetes. By relaxing these limitations, high-fat diet expanded diversity. As a result, the effect of cohousing—which had ameliorated the impact of STAT on weight during chow—faded in high-fat diet and the association between STAT and heavier weight returned. A plausible interpretation is that the switch to high-fat diet amplified earlier effects of STAT on the microbiota community, which were less obvious during chow and only manifested with the high-fat diet stress. That finding is supported by the observation—obtained by supervised machine learning—that the microbial taxa most strongly associated with weight differed in the chow and high-fat diet periods. An important conclusion from these analyses is that the fundamental metabolic “tone” is established in early life (first 4 weeks in a mouse), and that later restoration of a more normal microbiota that ameliorates the ecological changes is not sufficient to overcome the early life programing of metabolism by an altered microbiota.

We also saw that STAT affected females more than males, a difference which had been noted previously [21]. Sex disparities are common in microbiota studies in humans [33, 34] and mice [35–37]. If our results translate to humans, the susceptibility to weight gains after antibiotic exposure could reflect host differences, and help explain why obesity is more prevalent in women compared to men [38]. More generally, our results emphasize the importance of sex as a biological variable in microbiome studies.

The metabolite profiles that we obtained for a subset of the mice helped us understand the development of metabolic disease in the mouse STAT model. A prior study on lifetime exposure to low-dose (STAT) antibiotics had reported insulin resistance and non-alcoholic fatty liver disease [27]. Here, we found no evidence of differential insulin intolerance or liver disease in this limited early-life STAT model suggesting that relatively short-term STAT alone is insufficient to cause other aspects of metabolic syndrome, even with high-fat diet. The serum metabolites measured just before the switch from chow to high-fat diet revealed cellobiose, a disaccharide byproduct of cellulose degradation that is rapidly host-absorbed in experimental inflammatory bowel disease (IBD) [39]; its increase in the serum of mice that later gained more weight suggests that increased permeability might play a role in growth, or alternatively reflect particular microbiota compositions. Furthermore, although serum alpha-tocopherol (vitamin E) levels have been inversely associated with obesity in humans [40, 41], the increased levels in STAT and Control cohoused mice that we found compared to Control non-cohoused mice may reflect the differential physiologic state at the time the serum was obtained, before high-fat diet, rather than the large weight increases after high-fat diet. Since vitamin E is fat soluble, an early increase could predict future weight gains during high-fat diets. Increases in citric acid, 2-hydroxybutanoic acid, and phenylalanine in both obese humans [42, 43] and in this study may participate in the increased growth.

Our results revealed several new features of how early-life STAT and cohousing influence the microbiota. First, the α-diversity was unaffected by STAT and any impact of STAT and cohousing on weight gain was due to differences in community composition, which were captured—at least partially—along PCo2 in our β-diversity analysis. Second, we detected compositional differences in the microbiota of STAT and Control mice even after antibiotics stopped at week 4. In our previous study, the community had recovered more completely after stopping antibiotics [21]. STAT affected relatively minor components of the microbiota, such as *Akkermansia* and Proteobacteria, but not the ratio between the two major phyla—Bacteroidetes and Firmicutes—which dominated the microbiota during chow and high-fat diets.

The compositional differences described here compared with previous studies are consistent with the results seen in humans that microbiota resilience after disturbance can vary substantially [44] and outlast the influence of diet despite its rapid and reproducible effects on the microbiota composition [45]. Nonetheless, the use of an ecology framework, based on β-diversity metrics which ignore the identity of the specific microbial taxa, showed that the changes induced by diet dominated over the effect of cohousing. Identifying candidate microbes complemented these insights. For example, bacteria of the genus *Lactobacillus* have been associated with weight gain in mice [46], poultry [47], and newborn infants [48]. Nevertheless, the picture we revealed suggests complex dynamics: *Lactobacillus* initially decreased in STAT mice, but later increased when the mice switched to high-fat diet, and had opposite associations with weight during these periods while not being affected by cohousing. This highlights the importance of ecological quantities such as α- and β-diversity to clarify trends from these complex scenarios.

Finally, this study strengthens our confidence that antibiotics—given in a critical window in early life—can disrupt the microbiota and set the host on a resilient trajectory for greater weight later in life. Amidst reproducibility crises in medical sciences [49, 50], our study helps establish a firm causal link between the early-life microbiome perturbation induced by STAT and excessive weight [21]—a complex host phenotype which can depend on multiple factors. Our cohousing experiment with mice shows that attempting to change the microbiota composition after this early-life period has an impact, but its effects can vary between hosts and with diet.

This conclusion may be important for humans as well. Our gut microbiota changes rapidly when we are exposed to different microbes while traveling, when we change diet, when we get sick and take medications [29, 45]. The future of microbiota restoration to solve obesity problems and beyond may require personalization [51–54] that takes these complexities into account.

## Methods

### Animals

All animal experiments were performed according to an IACUC-approved protocol at NYUSoM. Mice were housed in a specific pathogen-free environment with a 12-h light/dark cycle. Six-week-old C57BL/6 mice (Jackson Laboratories, Bar Harbor, ME) were allowed to acclimate to our animal facility for 1 week before breeding. After 5 days, breeders were separated, and pregnant dams were randomized into treatment groups. Half the pregnant mothers were given low-dose penicillin G in their drinking water (STAT), as described [20]. Control mice did not receive antibiotics. STAT mice received 1 mg/kg body weight per day, which is in the middle of the suggested range published by the FDA for use of antibiotics for growth promotion in farm animals and well below the therapeutic dose for mice [55]. The mice were weaned at 28 days of life, at which point the antibiotic exposure ended in the STAT mice. Within 3 days of weaning, the litters were separated by sex and cages were randomly assigned. Cohoused cages each had two Control pups and two STAT pups. All mice were given normal chow after weaning (Purina Mills International Diet 5001, 13.5% kcal from fat) and switched to a 45% high-fat diet at week 16 of life (Research Diets D12451, 45% kcal from fat).

### Body composition

Beginning at 4 weeks of age and repeating every 4 weeks for the length of the experiment, a Lunar PIXImus II mouse densitometer (GE Medical Systems, Waukesha, WI) was used to determine body composition by dual energy X-ray absorptiometry while mice were under anesthesia by inhaled isoflurane, as described [20, 21].

### Analysis and modeling

We modeled the weekly measured scale weights (log-transformed) using linear mixed models. Treatment arm and cohousing were included as fixed effects, and categorical day and sex were included as random effects. The *fitlme* function of Matlab (2016a) was used to perform the tests and calculate the estimates. We also used Matlab to investigate α-diversity, β-diversity, and the impact of cohousing in the intestinal microbiota composition, fitting the equation *similarity* ~ *Cage* + *Litter* using for each time point using Matlab’s *fitlm* function. The supervised machine learning was implemented using the log-transformed weights, *z*-scored for each sex and each day, as the dependent variable. The microbiota compositions were also *z*-scored (after adding a pseudocount for missing values) for each sex and day, and used as the independent variables. The elastic net was carried out using the *lasso* function in Matlab using options cv = 5, alpha = 0.95 and mcreps = 10. The cross-validation partitions were stratified using function *cvpartition* with day, sex, and the cohousing/treatment as groups to ensure even representation in all training/testing iteration.

### Glucose and insulin tolerance

Mice were fasted for 4 h before performing glucose tolerance tests (GTT) and insulin tolerance tests (ITT). GTT: mice received an intraperitoneal injection of 1 mg glucose/g body weight. Glucose had been reconstituted in sterile water. Blood glucose was measured with an Abbott Freestyle Lite glucometer (Abbott Laboratories, Abbott Park IL) before injection (0 min) and after (15, 30, 60, and 120 min). The Abbott Freestyle Lite glucometer’s limits of detection are 20–500 mg/dL. When the glucometer registered values as “hi” or “low” they were plotted as being at the limit of detection, 500 or 20 mg/dL, respectively. ITT: mice received an intraperitoneal injection of 0.5 U/g body weight of insulin (Humulin R, Eli Lilly, Indianapolis IN) and glucose was measured as for GTT. Between 60–120 min after insulin injection, all females became severely hypoglycemic and had to be resuscitated with an injection of glucose and returned to their cages with food for observation.

### Metabolomics

Blood was collected by cardiac puncture, serum was separated by centrifugation at 3000 × *g* for 10 min at 4 °C, then stored at −80 °C. Serum samples (30 µL) were extracted with 1 mL cold 3:3:2 acetonitrile:isopropanol:water, mixed and centrifuged for 4 min at 14000 rpm, and the sample supernatants (450 µL) were dried, and reconstituted in cold 1:1 acetonitrile:water (450 µL). In addition, a total pool quality control (QC) sample was created by combining aliquots of equal volume from each serum sample, processed in the same way, and replicates prepared. Polar molecular functionalities were derivatized with methoxyamine hydrochloride (MeOX) in pyridine (40 mg MeOX/mL pyridine) for 90 min at 30 °C followed by *N*-methyl-*N*-(trimethylsilyl)-trifluoroactamide (MSTFA) for 45 min at 70 °C on an Eppendorf thermomixer at 1400 rpm. Fatty acid methyl ester standards were added to the extract to facilitate Retention Index calculation and quality control prior to analysis by gas chromatograph-time-of-flight mass spectrometry (GCTOF-MS). Individual study samples were randomized with total pool QC samples interspersed throughout the sequence. Samples were analyzed by injection onto an Agilent Technologies 7890A GC oven with a Leco Pegasus IV TOF MS. Data were collected using Leco ChromaTOF 4.51 software. Compound separation was achieved using a Restek Rxi®-5Sil MS (30 m × 0.25 mm internal diameter, and 0.25 µm in film thickness) capillary column. Electron Impact ionization mode at 70 eV was used with a scanning rate of 20 spectra per second over a mass range of 50–750 amu, and a transfer line temperature set to 280 °C. The ion source temperature was set to 250 °C. BinBase software (UC Davis, Davis CA) was used to align and assign peaks across samples [56, 57]. Multivariate analysis was accomplished using SIMCA 14.1 (Umetrics, Umeå, Sweden). Principal component analysis was used to demonstrate that the total QC pool samples tightly clustered in the center of the samples from which they were derived. Supervised multivariate analysis, OPLS-DA (orthogonal partial least squares discriminant analysis), was used to determine the signals most important to the differentiation of the four study groups.

### Hepatic lipid extraction and measurement

Lipids were extracted and measured from hepatic tissue, as described [27]. Briefly, 100 mg tissue was homogenized in 500 μl phosphate-buffered saline (PBS) with stainless beads for 1 min in a Powerlyzer homogenizer. Fifty microliters was removed for total protein measurement (BCA reagent; Thermo Scientific, Waltham, MA, USA) and 1.5 mL of 2:1 chloroform:methanol was added to the remaining solution, which was then vortexed and centrifuged for 10 min at 3000 rpm at 4 °C. The organic phase was collected and dried under nitrogen gas then dissolved in 500 μl 2% Triton-X 100 in chloroform, dried again, and dissolved in 100 μl PBS. Triglyceride and total cholesterol were quantitated with the Thermo Scientific Infinity Assays while free fatty acids were measured with the Wako NEFA kit (Wako Life Sciences, Richmond, VA, USA). Lipid mass was normalized to tissue mass and total protein mass giving similar results, only results normalized to tissue mass are displayed.

### Microbial community analysis

DNA was extracted from frozen samples using the 96-well MO BIO PowerSoil DNA Isolation Kit (MO BIO, Carlsbad CA). Barcoded fusion primers targeting the V4 region were used to amplify the 16S rRNA gene, as described [58]. Amplicon pools were sequenced on the Illumina Miseq platform with 150 bp paired-end reads. The QIIME pipeline [59] was used for downstream analysis. Fastq-join from EA-utils [60] was used to join paired-end reads with a minimum overlap of 30 base pairs, retaining only reads with perfectly matched paired-ends. Sequences were demultiplexed and quality filtered with QIIME. If a read had more than three consecutive low-quality bases (phred *q* score ≤20) it would be truncated, and any read that ended up being <75% of its original length was discarded. Sequences were assigned taxonomy with the open reference method in QIIME using UCLUST [61] and the Green Genes 2013 May database release as a reference [62]. Samples with <1800 reads were removed from the dataset. To reduce the number of features, the dataset was filtered to exclude any taxon that accounted for <0.01% of the total observation count. This dataset then was used to generate relative abundance plots, calculate α-diversity, β-diversity, and supervised machine learning in Matlab.

## Supplementary information

Supplemental figures
