# Supplementary material for: The impact of early-life sub-therapeutic antibiotic treatment (STAT) on excessive weight is robust despite transfer of intestinal microbes
Source: ISME J. 2019 Jan 16;13(5):1280–92. doi: 10.1038/s41396-019-0349-4 (PMC6474226; doi:10.1038/s41396-019-0349-4)
Supplement: Supplementary file 1 — Supplemental figures [file 41396_2019_349_MOESM1_ESM.docx]

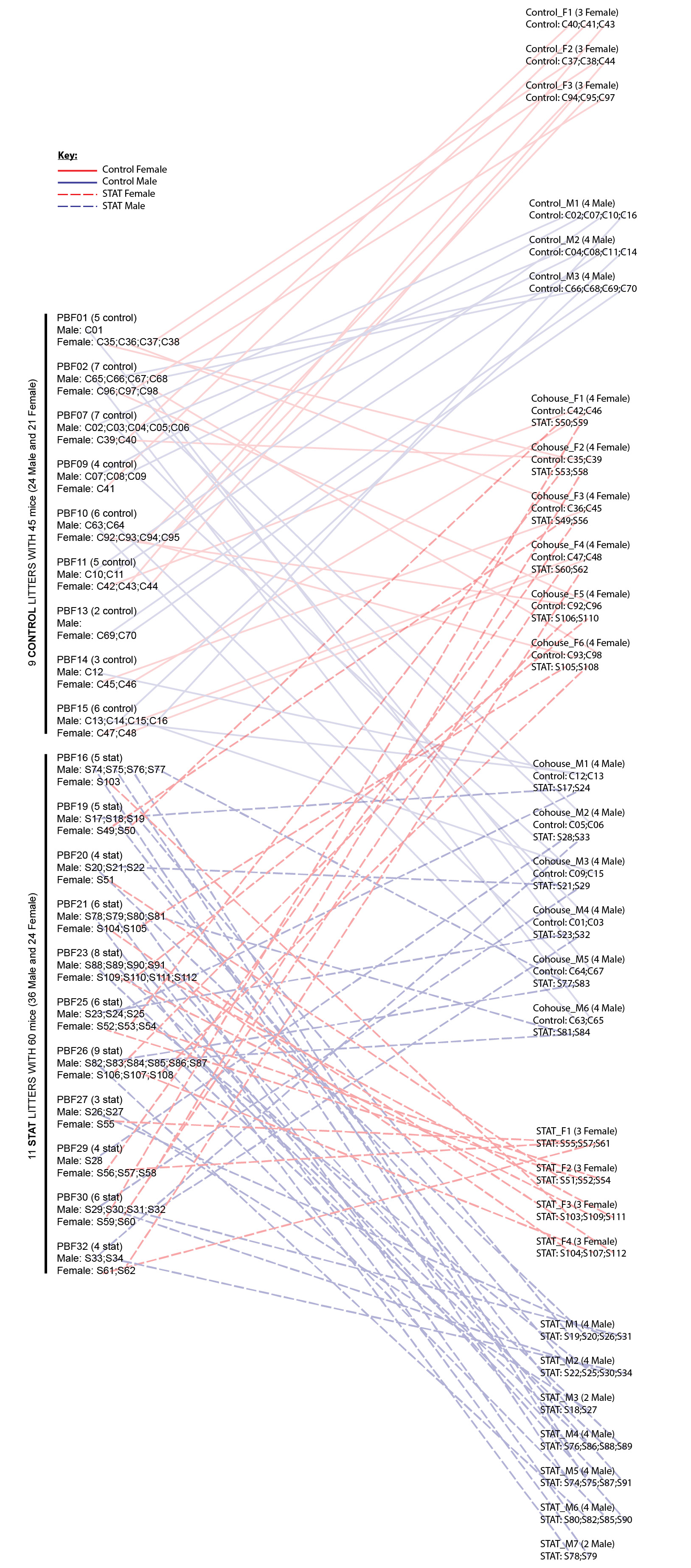


**Fig S1. Schematic of the cohousing scheme for the full experiment using 105 mice.**


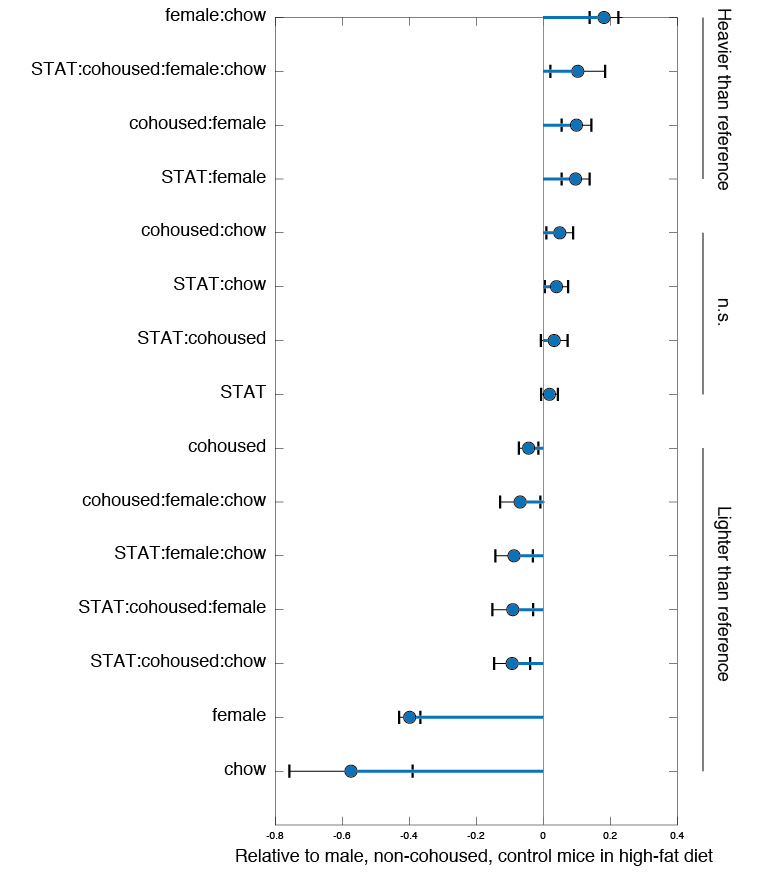


**Fig S2. The effect of STAT and cohousing are stronger in females than in males.** Mixed effects model correcting for day of life compared the effects of STAT, cohousing, diet and sex and the interactions between these variables (STAT*cohoused*female*chow). The model used male, non-cohoused, control mice during high-fat diet as the reference group. The interaction term STAT:cohoused:female:chow was significant (P=0.017).

**
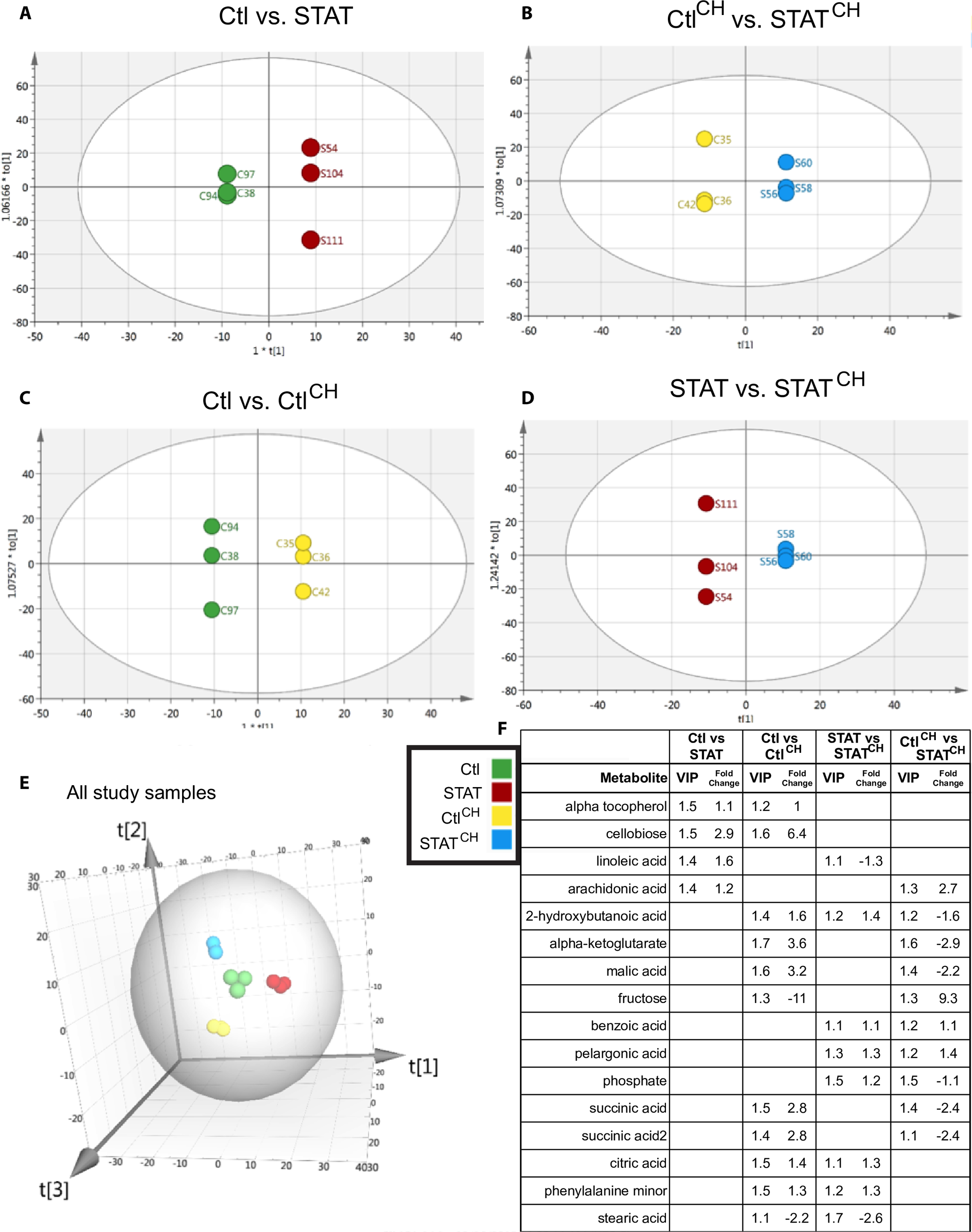
**

**Fig S3. Metabolomics of all four groups of female mice at week 15.** (Panels A-D) Score plots of OPLS-DA analysis of serum samples show separation between each pair of groups compared. The statistics for the model fits of the OPLS-DA: (A) R2X = 0.906, R2Y = 1, Q2 = 0.952, with R: goodness of fits, Q: goodness of prediction. (B) R2X = 1, R2Y = 1, Q2 = 1 (C) R2X = 0.871, R2Y = 1, Q2 = 0.991 (D) R2X = 1, R2Y = 1, Q2 = 1 (E) Metabolomic profiles in serum samples of Ctl (Control), STAT, CtlCH and STATCH female mice at week 15 of life. OPLS-DA analysis of all groups with 3 predictive components shows separation between all 4 groups. The statistics for the model: R2X = 0.673, R2Y = 0.955, Q2 = 0.176. (F) List of known metabolites important for separating pairs of groups (Variable Influence on Projection (VIP)≥1). For each comparison, positive fold-change indicates median of the last group listed in the column label is greater than the median of the first group listed in the column label.

**
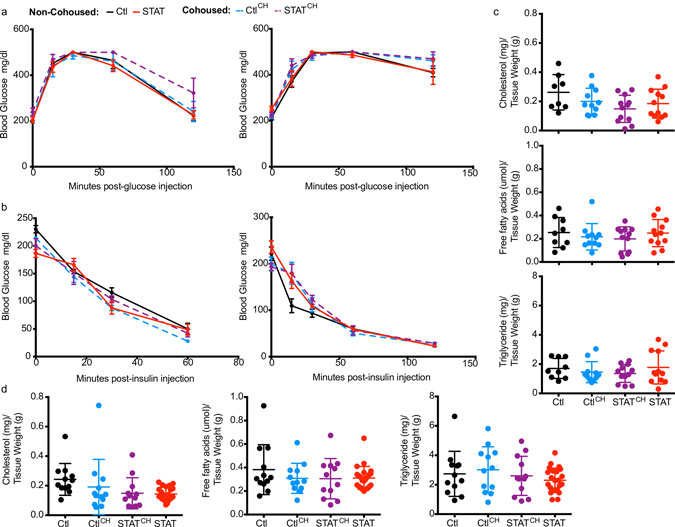
**

**Fig S4. Early-life STAT did not impact later glucose homeostasis or hepatic lipid accumulation.** (a-b) For glucose and insulin tolerance testing of 31-week-old male and female mice, 4 mice from each group were challenged with 5g/kg dextrose, or with 0.5 U/kg human insulin by intraperitoneal injection. Blood glucose was measured by glucometer at 0, 15, 30, 60, and 120 min post-injection. Figures show mean ± SEM (a) Glucose tolerance test results for female (left) and male (right) mice. (b) Insulin tolerance test results for female (left) and male (right) mice. (c-d) Lipids were extracted from frozen livers, quantified, and normalized to tissue weight. All values (mean ± SD) are shown for cholesterol, free fatty acids, and triglycerides for (c) female and (d) male mice.


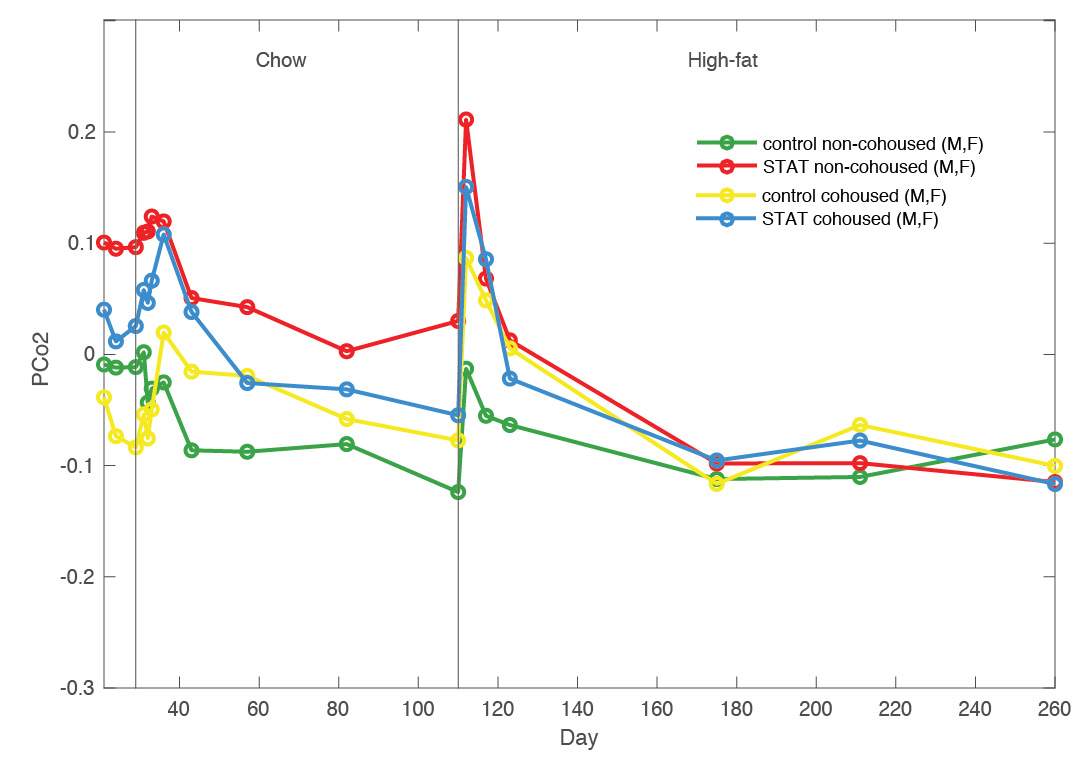


**Fig S5. Overlay of the four groups presented in Fig. 5B and Fig. 5C only depicting the lines for the averages.** With less data displayed in the background, this figure shows more clearly that co-housed control mice have higher PCo2 scores at most time-points than their non-cohoused counterparts.
